# The Binding Affinity and Molecular Basis of the Structure-Binding Relationship between Urinary Tamm-Horsfall Glycoprotein and Tumor Necrosis Factor-α

**DOI:** 10.3390/molecules171011978

**Published:** 2012-10-11

**Authors:** Cheng-Han Wu, Ko-Jen Li, Sue-Cien Siao, Yu-Hsuan Chen, Tsai-Hung Wu, Chang-Youh Tsai, Chia-Li Yu

**Affiliations:** 1 Institute of Clinical Medicine, National Taiwan University College of Medicine and National Taiwan University Hospital, No.7 Chung-Shan South Road, Taipei 100, Taiwan; 2 Institute of Clinical medicine, National Yang-Ming University College of Medicine, No.155 Li-Nong Street, Shih-Pai, Taipei 11217, Taiwan; 3 Institute of Molecular Medicine, National Taiwan University College of Medicine, No.7 Chung-Shan South Road, Taipei 100, Taiwan; 4 Section of Nephrology, Taipei Veterans General Hospital, No.201 Section 2, Shih-Pai Road, Taipei 11217, Taiwan; 5 Section of Allergy, Immunology and Rheumatology, Taipei Veterans General Hospital, No.201 Section 2, Shih-Pai Road, Taipei 11217, Taiwan

**Keywords:** Tamm-Horsfall glycoprotein, tumor necrosis factor-α, binding affinity, structure-binding relationship, glucosamine-containing mannose

## Abstract

In a previous study we noted significant THP binding to TNF-α, but did not explore the molecular basis of the structure-binding relationship. In this study, we used lectin-binding ELISA to assess the carbohydrate compositions of THP, BSA, IgG, TNF-α, and IFN-γ. We identified β(1,4)-*N*-acetylglucosamine oligomers (GlcNAc) and GlcNAc/branched mannose in BSA, IgG, TNF-α, and THP, but not in IFN-γ. These carbohydrate moieties mediated binding with THP. Small amounts of Siaα(2,3)Gal/ GalNAc, Sia(2,6)Gal/GalNAc, and mannose residues were also present in THP and TNF-α. Binding affinity (K_d_) between THP and TNF-α by Scatchard plot analysis was 1.4–1.7 × 10^−6^ M, lower than antigen-antibody or ligand-receptor binding affinities. To elucidate the structure-binding relationship of THP-TNF-α, THP was digested with neuraminidase, β-galactosidase, *O*-sialoglycoprotein endopeptidase, carboxypeptidase Y, or proteinase K. β-galactosidase increased binding capacity of THP for TNF-α. Monosaccharide inhibition suggested that α-methyl-D-mannoside, GlcNAc, and GalNAc, but not sialic acid, suppress THP-TNF-α binding as detected by ELISA. We conclude that sugar-lectin and sugar-protein interactions between cognate sites in THP and TNF-α mediate their binding.

## 1. Introduction

Tamm-Horsfall glycoprotein (THP), a 80–90 kDa macromolecule, is produced by the mammalian renal thick ascending limb of Henle’s loop [1,2]. THP is a crucial defense protein, protecting the kidney and urinary tract from microbial invasion [3,4]. Carbohydrate analysis of THP revealed the unique macromolecule contains approximately 25%–35% complex carbohydrate side-chains, particularly sialic acid [5,6,7]. These florid sugar side-chains render THP capable of binding with a number of soluble protein molecules [8,9,10,11,12,13,14] and surface-expressed molecules on neutrophils [15,16,17], lymphocytes [16], monocytes/macrophages [18], and renal glomerular mesangial cells [16]. THP binding to the cell surface activates cellular functions [11,17,19]. We previously demonstrated that the binding capacity of THP for tumor-necrosis factor (TNF-α) was higher than it is for bovine serum albumin (BSA), human IgG, human complement component 1q (C1q), interleukin (IL) 8 (IL-8), or IL-6 [11]. 

TNF-α is a 17-kDa glycoprotein containing lectin-like domains capable of binding with the glycomoiety of THP [14]. Many authors have reported that carbohydrate side chains in THP consist mainly of N-linked glycans with high mannose sequences carried by Asn251—Man6GlcNAc_2_ and Man5GluNAc_2_ that mediate interactions with type-1 fimbriated *Escherichia coli* [20,21,22,23]. O-linked chains in THP molecules are responsible for interactions with different proteins [24]. TNF-α may play an important role in chronic inflammatory diseases such as rheumatoid arthritis [25,26]. Hession *et al.* [27] found that THP might act as a unique renal regulatory glycoprotein via binding to a number of potent circulatory cytokines including TNF-α and IL-1β. In clinical practice, anti-TNF-α biological therapy could rapidly suppress rheumatoid activity better than conventional disease-modified anti-rheumatic drugs (DMARDs) therapy [28,29]. However, the molecular basis of the structure-binding relationship between THP and TNF-α remains unclear. In this study, we explored the binding-structure foundation by using lectin-binding ELISA, enzyme digestion, and monosaccharide inhibition tests. These findings may yield a novel therapeutic strategy for rheumatoid arthritis.

## 2. Results and Discussion

### 2.1. Non-Specific THP Binding to Serum Proteins and Proinflammatory Cytokines

**Figure 1:**
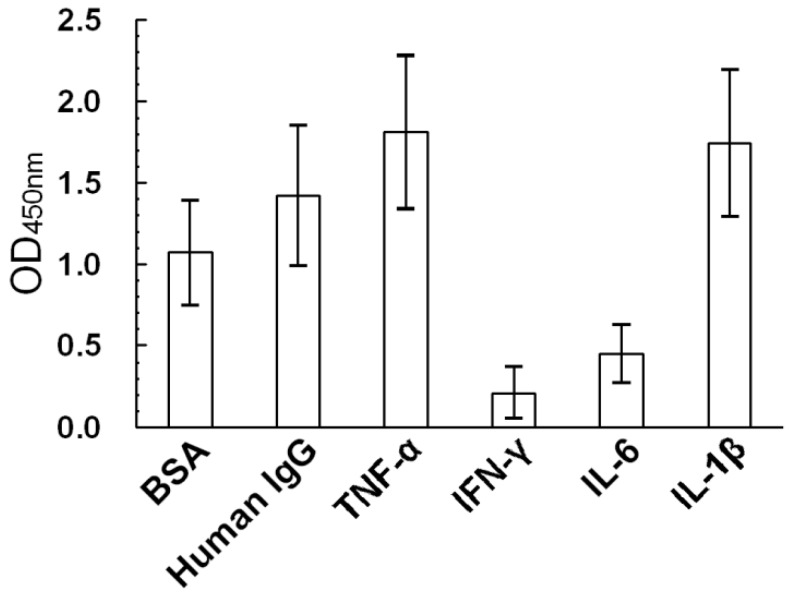
Binding capacity of THP (10 μg/mL) for different proteins including bovine serum albumin (BSA), human IgG, tumor necrosis factor-α (TNF-α), gamma-interferon (IFN-γ), interleukin 6 (IL-6), and interleukin 1β (IL-1β) by ELISA.

THP binds non-specifically to a broad spectrum of protein molecules [11,16]. To confirm this property, microwells were coated with 100 μL of 20 μg/mL BSA, human IgG, human recombinant TNF-α, IFN-γ, IL-6, or IL-1β and incubated at 37 °C for 2 h and 4 °C overnight. THP (100 μL at 10 μg/mL) was then added to the microwells and incubated at 37 °C for 2 h. HRP-conjugated anti-uromucoid antibodies were added to detect THP binding to different proteins. We thus confirmed THP purified from normal human urine non-specifically bound different protein molecules with diverse capacities (Figure 1). Maximum binding occurred between THP and TNF-α; minimum binding occurred between THP and IFN-γ. 

### 2.2. Dose-Dependent Binding Between THP and TNF-α

**Figure 2:**
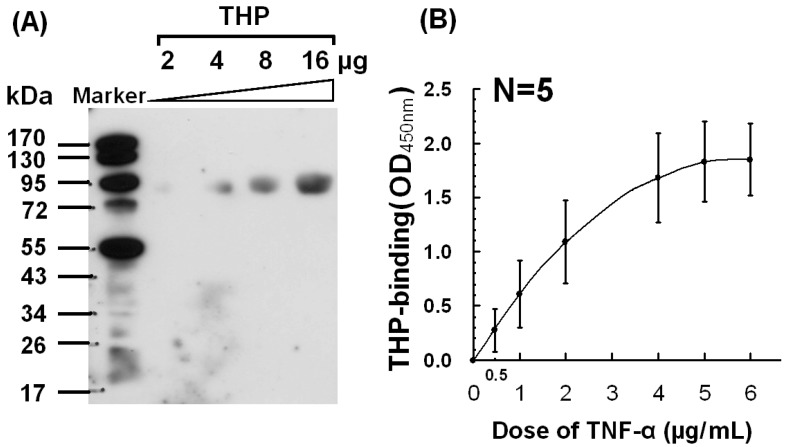
Dose-responsive binding of THP with human recombinant TNF-α detected by (**A**) Western blot, and (**B**) ELISA.

To verify binding between THP and TNF-α, Western blots (2 to 16 μg/mL THP; Figure 2A) and ELISA (0.5 to 6 μg/mL TNF-α; Figure 2B) were performed. 

Binding between THP and TNF-α was clearly dose-dependent. Moonen *et al.* [30] argued that native TNF-α and IL-1β do not bind THP because TNF-α in liquid phase does not competitively inhibit THP binding to TNF-α-coated microtiter plates. However, Hession *et al.* [27] argued that THP is a renal ligand for cytokines *in vivo*. Why THP only reacts with denatured, not native, TNF-α, and whether TNF-α and IL-1β are denatured *in vivo* requires further investigation. 

### 2.3. Low Binding Affinity with K_d_ =1.4 −1.7 × 10−^6^ M between THP and TNF-α

**Figure 3:**
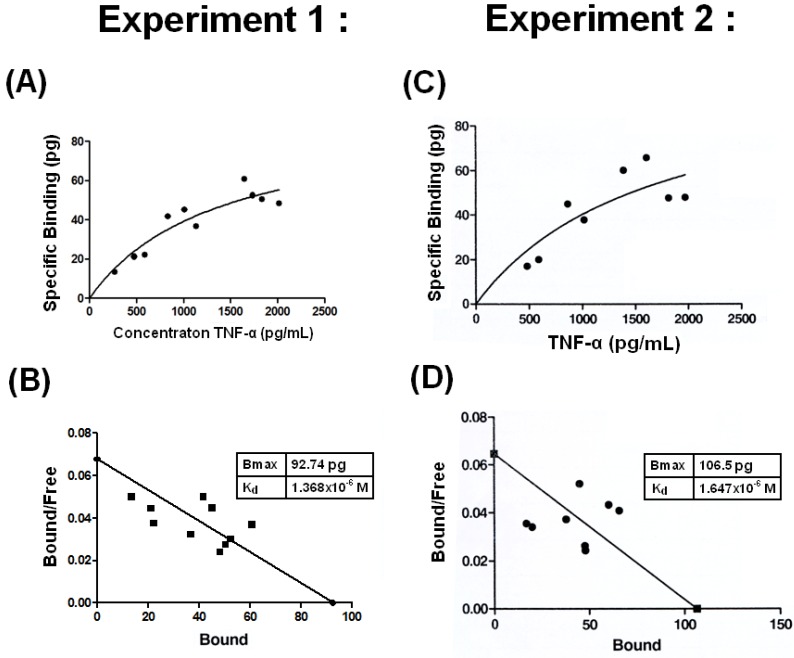
Binding affinity of THP for TNF-α was conducted in two independent experiments by ELISA. (**A**) and (**C**): The specific binding *vs*. TNF-α concentrations are plotted. (**B**) and (**D**): Y transformed curves are derived from the specific bindings of the two experiments. Bmax and K_d_ values were then calculated by Scatchard plot analysis as described in Materials and Methods in Section 3.6.

It is believed that the binding affinity between two proteins is less than antigen-antibody or ligand-receptor interactions. We estimated the THP-TNF-α binding affinity as described by Katanick *et al.* [31] with some modifications. The specific binding *vs.* TNF-α concentrations in two experiments are plotted in Figure 3A (Experiment 1) and 3C (Experiment 2). 

The Y transformed curves derived from the specific binding data are plotted in 3B (Experiment 1) and 3D (Experiment 2). B_max_ and K_d_ values were calculated by Scatchard plot analysis shown in Figure 3. The K_d_ values of the two experiments were 1.368 × 10^−6^ M (Figure 3B) and 1.647 × 10^−6^ M (Figure 3D). However, Rhodes *et al.* [32] demonstrated two binding affinities between sheep THP and IgG; a high-affinity K_d_ of 10^−8^–10^−9^ M and a low-binding affinity K_d_ of 10^−6^–10^−7^ M. Although we did not measure the K_d_ of human THP-IgG binding, it was 20% lower than human THP-TNF-α binding as shown in the ELISA results (Figure 1). Experiments to measure the binding affinity of different mammalian THPs with human TNF-α are underway.

### 2.4. Carbohydrate Compositions of THP and THP-Binding Proteins

**Figure 4:**
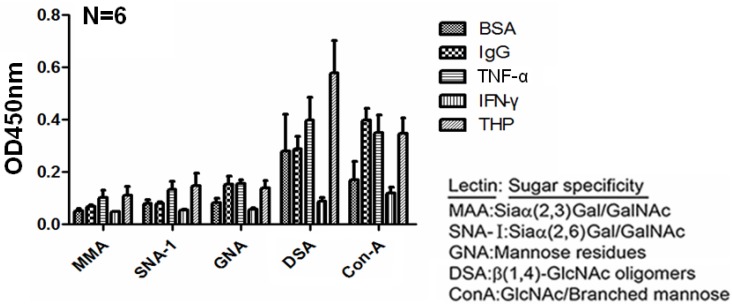
Carbohydrate compositions of BSA, human IgG, human recombinant TNF-α, human recombinant IFN-γ, and human urinary THP were dissected by lectin-binding assay using 5 lectins with different sugar-binding specificities.

Sherblom *et al.* [14] demonstrated a lectin-like interaction between human recombinant TNF-α and uromodulin. We hypothesized that THP molecules may play dual roles in protein binding as their high carbohydrate content (25%–30%) are targets of lectin-like domains of TNF-α. Conversely, some THP domain structures such as the *zona pellucina* (ZP) domain may possess adhesive molecule-like or even lectin-like properties and bind to carbohydrate components in TNF-α molecules. We assessed the carbohydrate compositions of THP and THP-binding molecules. Lectin-binding ELISA method was performed with 5 lectins with specific sugar moieties to detect the sugar compositions of BSA, IgG, TNF-α, IFN-γ, and THP (Figure 4). IFN-γ contained minimal glycomoieties, consistent with its minimal binding to THP. We noted that BSA, IgG, and TNF-α contain abundant β(1,4)-GlcNAc oligomers and GlcNAc/branched mannose. These glycomoieties are also present in THP. Muchmore *et al.* [33] found that high-mannose glycopeptides [Man5(6)GluNAc_2_-Asn] THP interact with recombinant TNF-α and IL-1β. It is possible the high-mannose glycans in human THP are carried by Asn251 [21,23]. IL-2 also exhibits lectin-like properties specific for high-mannose glycopeptides capable of binding THP [13,33]. Lucas *et al.* [34] demonstrated that trypanosome-TNF-α interaction was inhibited by N,N′-diacetylchitobiose. This domain structure also possessed lectin-like affinity for TNF-α. These results suggest that sugar-lectin-sugar interactions are one of the mechanisms for THP-protein binding.

### 2.5. β-galactosidase-digested THP Enhanced THP-TNF-α Binding

**Figure 5:**
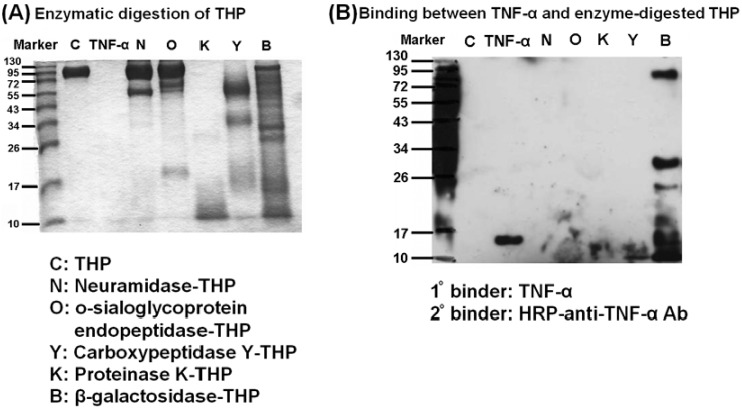
Binding capacity of intact THP and enzyme-digested products with TNF-α, was detected by Western blotting. (**A**) The molecular size of intact THP, TNF-α, and the different enzyme-digested THP products was identified by Coomasie blue staining. (**B**) The binding capacity of intact THP and enzyme-digested THP products was detected by Western blot using TNF-α (1° binder) and HRP-anti-TNF-α antibody (2° binder) as indicators.

THP contains complex carbohydrate side chains around the protein core structure; thus, it is necessary to determine whether the side chain glycomoiety or protein-core structure mediates THP-TNF-α binding. We used carbohydrate-degrading enzymes (neuraminidase, β-galactosidase), protein-degrading enzyme (proteinase K), and glycoprotein-degrading enzymes (*O*-sialoglycoprotein endopeptidase, carboxy- peptidase Y) to digest intact THP as shown in Figure 5A. Digested THP products were then immunoblotted with TNF-α (1° binder) and HRP-anti-TNF antibody (2° binder). As shown in Figure 5B, only β-galactosidase-digested THP products reacted with TNF-α on Western blots. Thus, galactoside residues in the THP side chain prevent THP-TNF-α binding. Because the amount of THP (4 μg) used in Figure 5 was insufficient for reacting with TNF-α as shown in Figure 2, it is difficult to conclude whether the protein-core structure is involved in THP-TNF-α binding. We suggest the ZP domain responsible for polymerization of THP into supra-molecular structures mediates homologous THP-THP binding and heterogeneous THP-TNF-α binding. Genetic manipulation to delete the ZP domain is in progress.

### 2.6. α-Methyl-D-mannoside (α-MDM), N-acetyl-galactosamine, and N-acetyl-glucosamine Mediate THP-TNF-α Binding

**Figure 6:**
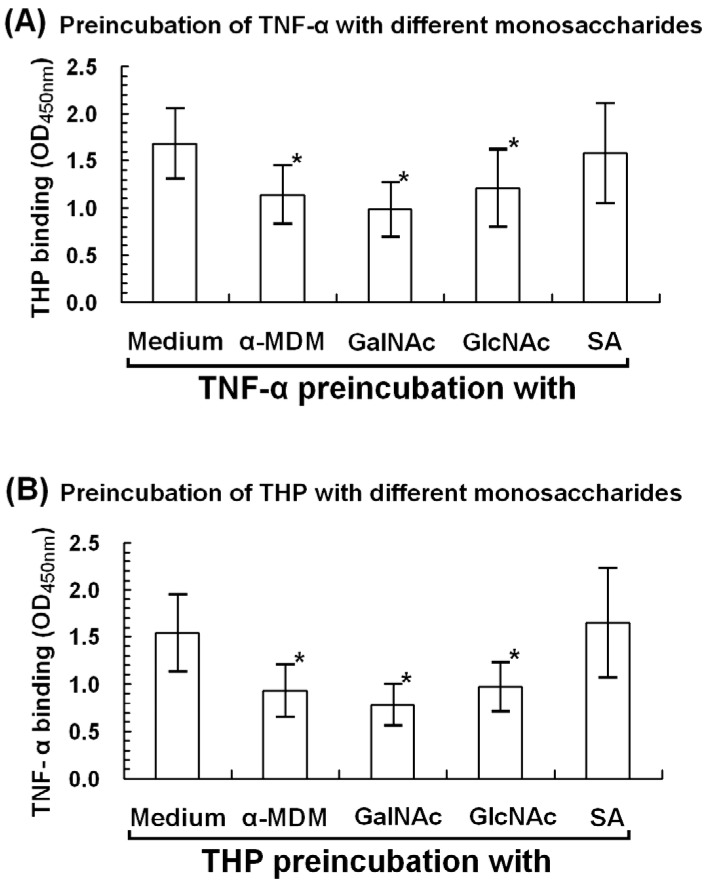
Monosaccharide inhibition assay was conducted to identify the carbohydrate(s) mediating THP and TNF-α binding. (**A**) TNF-α-precoated microcells were incubated with α-methyl-D-mannoside (α-MDM), N-acetyl-galactosamine (GalNAc), *N*-acetyl-glucosamine (GlcNAc), or sialic acid (SA) for 2 h, incubated with THP, and detected by ELISA (**B**) THP-precoated microwells were incubated with monosaccharides for 2 h, incubated with TNF-α, and detected by ELISA. * denotes *p* < 0.05.

Siaα(2,3)Gal/GalNAc, Siaα(2,6)Gal/GalNAc, β(1,4)-GalNAc oligomers, mannose residues, and GlcNAc/branched mannose are present in THP and TNF-α (Figure 4). To determine whether these common monosaccharides (sialic acid, mannoside, glucosamine, and galactosamine) are also involved in THP-TNF-α binding, we preincubated each monosaccharide with either TNF-α or THP and then added the other molecule. As demonstrated in Figure 6, mannose, GlcNAc, and GalNAc significantly suppressed THP-TNF-α binding. These results are consistent with Sherblom *et al.* [13,34] in which high mannose-containing glycopeptides such as Man5GlcNAc2-R and Man6Glc-NAc2-R mediate THP-TNF-α binding through sugar-lectin-sugar interactions. In contrast, sialic acid did not influence protein-protein binding, as reported by Parsons *et al.* [7] and Huang *et al.* [35]. These results support our central theme that both sugar-lectin and sugar-protein interactions between cognate sites in THP and TNF-α mediate THP-TNF-α binding.

## 3. Experimental

### 3.1. Reagents and Antibodies

Purified human IgG, BSA, neuraminidase, β-galactosidase, proteinase K, and carboxypeptidase Y were purchased from Sigma-Aldrich (St. Louis, MO, USA). *O*-sialoglycoprotein endopeptidase was purchased from Cedar Lane Laboratories (Burlington, NC, USA). Biotin-conjugated lectins specific to unique carbohydrate moieties, including MMA [Siaα(2,3)Gal/GalNAc], SNA-1 [Siaα(2,6)Gal/ GalNAc], GNA [mannose], DSA [β(1,4)-GlcNAc oligomers], and ConA [GlcNAc/branched mannose] were obtained from E-Y Labs (San Mateo, CA, USA). Human recombinant TNF-α, IFN-γ, IL-6, and IL-1β were obtained from R&D Systems (Minneapolis, MN, USA). Mouse monoclonal anti-human TNF-α antibody and HRP-conjugated anti-TNF-α antibody were purchased from R&D Systems. α-Methyl-D-mannoside (α-MDM), *N*-acetyl-D-glycosamine (GlcNAc), *N*-acetyl-D-galactosamine (GalNAc) and *N*-acetylneuraminic acid (sialic acid, SA) were purchased from Sigma.

### 3.2. Purification of THP from Normal Human Urine

Twenty-four-hour urine was collected in a clean glass bottle from a normal individual. We followed the purification procedures described in our previous report [20]. THP purity and relative molecular weight were detected by 10% SDS-PAGE. Identification of THP was confirmed by Western blotting with anti-uromucoid antibody (The Binding Site Ltd, University of Birmingham Research Institute, Birmingham, UK). This study was approved by the IRB and Medical Ethics Committee, Taipei Veterans General Hospital, Taiwan (VGH IRB NO: 94-07-27A). All participants provided signed, informed consent.

### 3.3. THP Digestion by Carbohydrate-, Glycoprotein-, or Protein-Specific Enzymes

THP digestion was performed as described by Sherblom *et al.* [14]. All enzyme digestions were performed at 37 °C for 16–24 h. Final enzyme concentrations were: neuraminidase (10 U/mL) in 50 mM sodium acetate, pH 5.0; β-galactosidase (0.05 U/mL) in 50 mM sodium acetate, pH 5.0; *O*-sialoglycoprotein endopeptidase (50 μg/mL) in PBS, pH 7.2; carboxypeptidase Y (enzyme:substrate 1:10) in 0.2 M pyridine-acetate buffer, pH 7.2; proteinase K (0.5 μg/mL) in 10 mM Tris buffer, pH 7.5 with 1 mM MgCl2. The enzyme-cleaved THP products were then heated at 65 °C for 60 min to inactivate residual enzyme. The digested products were intensively dialyzed against alkaline distilled water, pH 9.0 for 24 h, changing the dialysate every 2 h to remove the products smaller than 10 kDa. The digested products were lyophilized and stored at −20 °C.

### 3.4. Binding Activity between Intact or Enzyme-Digested THP Products and TNF-α

#### 3.4.1. Western Blot

THP (2–16 μg) was separated by 10% SDS-PAGE. After transfer to a membrane, TNF-α (2 μg/mL) was added and incubated for 24 h at 4 °C, then probed with HRP-anti-TNF-α antibody.

#### 3.4.2. ELISA

THP (100 μL at 20 μg/mL) was placed in microtiter wells and incubated for 24 h at 4 °C. TNF-α (0.5~6.0 μg/mL) was added and incubated overnight at 4 °C. After several washes, HRP-conjugated anti-TNF-α antibody was added and incubated at room temperature for 2 h; color development and absorption were measured as OD_450_. 

### 3.5. Scatchard Plot Analysis and K_d_ Calculations

A Scatchard plot was used to analyze the binding affinities of THP and TNF-α. We followed the method described by Kananick *et al.* [31] with modifications: radioisotope was replaced by HRP-conjugated-TNF-α in the ELISA. Briefly, recombinant human TNF-α (0, 500, 1,000, 1,500, 2,000, and 2,500 pg/mL) were added to THP-coated microwells and incubated at room temperature for 2 h with continuous shaking. The mixture was spun at 450 *g* for 20 min. Unbound THF-α in the supernatant and thrice-washed aspirates were collected. Bound TNF-α in the microwells was lysed with 200 µL lysis buffer containing 50 mM borate, 150 mM NaCl, 1% NP-40, 0.5% sodium deoxycholate, and 25 mM phenylmethylsulphonyl fluoride, pH 8.0. Bound and unbound TNF-α were measured by ELISA. A Scatchard plot was drawn and analyzed by the “Prism” statistical program provided by GraphPad Software [36] to calculate the dissociation constants (K_d_). K_d_ is defined as the ratio of unbound and bound molecules at equilibrium: K_d_ = [A] × [B]/[AB]. Thus, small K_d_ indicates high-affinity interactions and large K_d_ values indicate low-affinity interactions.

### 3.6. Monosaccharide Inhibition of THP-TNF-α Binding

#### 3.6.1. Microwell-Coated TNF-α Was Preincubated with Different Monosaccharides

α-MDM, GalNAc, or GlcNAc (10 µg) was incubated in TNF-α-coated microwells overnight at 4 °C. THP (2 μg/well) was added and incubated overnight at 4 °C. HRP-anti-uromucoid antibody was added. After reaction at room temperature for 2 h, THP-TNF-α binding was measured as OD_450_.

#### 3.6.2. Microwell-Coated THP Was Preincubated with Different Monosaccharides

THP-coated microwells were preincubated with 10 μg of α-MDM, GalNAc, or GlcNAc overnight at 4 °C. TNF-α (2 μg/mL) was added and incubated overnight at 4 °C. HRP-anti-TNF-α antibody was added and incubated at room temperature. The binding of TNF-α to THP was detected by OD_450_ nm.

### 3.7. Statistical Analysis

Results are reported as mean ± S.D. Continuous variables were assessed by non-parametric Wilcoxon rank-sum test using commercially available software (Stata/SE8.0 Windows). Statistical significance was indicated by *p* < 0.05.

## 4. Conclusions

We have explored the molecular basis of the structure-binding relationship between THP and TNF-α. At least four original findings are reported here: (1) The K_d_ of THP-TNF-α binding was around 1.5 × 10^−6^ M, less than antigen-antibody or ligand-receptor binding. (2) THP and TNF-α contain high amounts of β(1,4)-GlcNAc oligomers and GlcNAc/branched mannose, which are relevant to THP-TNF-α binding. (3) Galactoside in THP carbohydrate side-chains hindered THP-TNF-α binding via sugar (in the THP molecule)-lectin (in the TNF-α molecule)-sugar (in the TNF-α molecule)-ZP domain (in the THP molecule) interactions. (4) Many monosaccharides such as α-methyl-D-mannoside, *N*-acetylglucosamine, or N-acetylgalactosamine, but not sialic acid, mediate THP-TNF-α binding. TNF-α is a crucial proinflammatory cytokine in many inflammatory disorders including rheumatoid arthritis, ankylosing spondylitis, psoriasis/psoriatic arthropathy, or inflammatory bowel diseases. Identification of the THP domain structure(s) responsible for TNF-α binding may yield a novel therapeutic strategy for acute and chronic inflammatory diseases.
